# A Review of Dengue’s Historical and Future Health Risk from a Changing Climate

**DOI:** 10.1007/s40572-021-00322-8

**Published:** 2021-07-16

**Authors:** Sutyajeet Soneja, Gina Tsarouchi, Darren Lumbroso, Dao Khanh Tung

**Affiliations:** 1 United Nations Development Programme, Vietnam Office, Washington, DC USA; 2 HR Wallingford, Howbery Park, Wallingford, Oxfordshire, OX10 8BA UK; 3 United Nations Development Programme, Vietnam Office, 304 Kim Ma, Hanoi, Vietnam

**Keywords:** Dengue, Climate change, Global health, Vector borne disease

## Abstract

**Purpose of review:**

The purpose of this review is to summarize research articles that provide risk estimates for the historical and future impact that climate change has had upon dengue published from 2007 through 2019.

**Recent findings:**

Findings from 30 studies on historical health estimates, with the majority of the studies conducted in Asia, emphasized the importance of temperature, precipitation, and relative humidity, as well as lag effects, when trying to understand how climate change can impact the risk of contracting dengue. Furthermore, 35 studies presented findings on future health risk based upon climate projection scenarios, with a third of them showcasing global level estimates and findings across the articles emphasizing the need to understand risk at a localized level as the impacts from climate change will be experienced inequitably across different geographies in the future.

**Summary:**

Dengue is one of the most rapidly spreading viral diseases in the world, with ~390 million people infected worldwide annually. Several factors have contributed towards its proliferation, including climate change. Multiple studies have previously been conducted examining the relationship between dengue and climate change, both from a historical and a future risk perspective. We searched the U.S. National Institute of Environmental Health (NIEHS) Climate Change and Health Portal for literature (spanning January 2007 to September 2019) providing historical and future health risk estimates of contracting dengue infection in relation to climate variables worldwide. With an overview of the evidence of the historical and future health risk posed by dengue from climate change across different regions of the world, this review article enables the research and policy community to understand where the knowledge gaps are and what areas need to be addressed in order to implement localized adaptation measures to mitigate the health risks posed by future dengue infection.

**Supplementary Information:**

The online version contains supplementary material available at 10.1007/s40572-021-00322-8.

## Introduction

According to the World Health Organization, an estimated 390 million (range 284–528 million) people worldwide are infected with dengue annually, 96 million (range 67–136 million) of which present clinical manifestations [1–3]. People in more than 125 countries, encompassing over 50% of the world’s population, are potentially at risk of infection [4], with the main vectors for transmission to humans being the *Aedes aegypti* and *Aedes albopictus* mosquitoes [5–7]. Caused by four closely related dengue viral serotypes (DENV 1-4) of the genus Flavivirus, dengue infection clinically manifests itself in many ways ranging from acute febrile illness, nausea, vomiting, eye/muscle/joint/bone pain, rashes, life-threatening situations (e.g., hemorrhage, known as dengue hemorrhagic fever), and even death with a case fatality ranging from lower than 1 to 20% [5, 8–12]. Furthermore, the global total direct (medical care and travel) and indirect (lost time and productivity) cost of dengue illness has been estimated at $8.9 billion (USD) annually [13]. Dengue is one of the most rapidly spreading viral diseases in the world, with the burden of disease having increased an estimated 30-fold over the last half century, despite increasing efforts to curb or reverse the upward trend [5, 14]. Many factors have contributed towards this spread including globalization, trade and shipping, shifts in demographics and urbanization patterns, inadequate domestic water supplies, and an increase of infected travelers acting as carriers over recent decades [5, 15, 16]. Weather or climate variables, such as temperature, humidity, high levels of precipitation, and vapor pressure have shown strong associations with altering the risk of contracting dengue [1, 8, 17]. Through multiple, interrelated mechanisms, climate variables can influence dengue transmission dynamics (e.g., by lengthening the dengue ‘season’ in endemic areas or stimulating the establishment of dengue in nascent areas), or even alter the temporal and spatial dynamics of dengue ecology (e.g., by increasing the mosquitoes flying range and shortening the incubation period) [8, 18–22]. Several studies have illustrated that climate change, via changes in temperature and precipitation, as well as increases in intensity, frequency, and duration of extreme weather events, has and will continue to impact the transmission of infectious diseases like dengue in many different parts of the world and especially in temperate regions [23–29].

Possessing an understanding of what epidemiological evidence currently exists on how climate change has historically impacted the risk of dengue infection, and how it may impact future risk, is important to understand so that future funding can be directed towards addressing knowledge gaps in order to better inform the development of localized health adaptation strategies. In this study, we synthesize recent literature assessing the historical and future health risk of dengue infections from climate change across all regions of the world.

## Methods

**Fig. 1:**
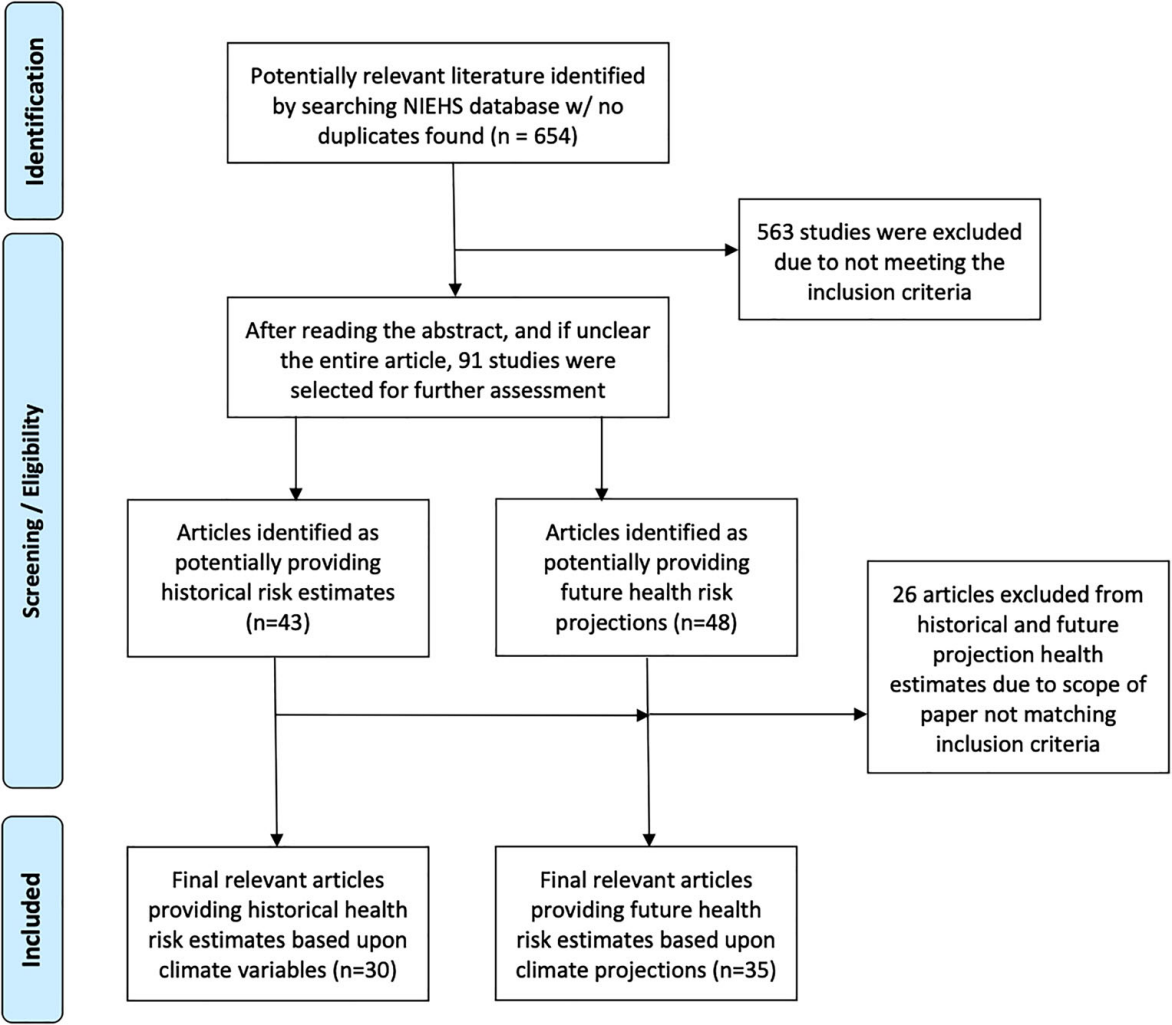
Flow chart illustrating article selection process for conducting literature search

From April to June 2020, we searched the U.S. National Institute of Environmental Health Sciences (NIEHS) Climate Change and Health Literature Portal [30], which maintains a database of literature related to climate change and health from January 2007 to September 2019. This date range represents the earliest allowable date and the most recent date that literature had been uploaded into the database, respectively. We utilized the keyword “dengue” and included studies published in English that provided epidemiological health risk estimates (e.g., relative risk or odds ratios) in relation to climate variables based upon historical dengue infection data or future climate projection scenarios, as well as changes in mosquito habitat, that specifically referenced changes to potential exposure for humans to dengue. Furthermore, we focused on health risk estimates that exposure to or contracting dengue instead of specific morbidity or mortality estimates given the multiple ways that dengue manifests itself as well as range of case fatality as previously mentioned. We excluded studies that were systematic or meta-analysis review articles, studies providing only information on model development (e.g., correlation coefficients), studies presenting risk across time periods (e.g., seasonality, El Niño–Southern Oscillation or ENSO) as a standalone but not quantitatively describing how these periods were changing as a result of climate change, studies providing a risk function only in graphic format with limited information on providing 95% confidence intervals (95%CI), and commentaries (see Figure 1 for flow chart of literature search strategy).

Study data, health and climate information, and key findings were extracted from each relevant paper and subsequently organized by continent, then alphabetical name of country, and chronological order of publication date. Studies providing historical health risk estimates are presented in Table 1 and studies presenting health risk estimates based upon future climate projection scenarios are presented in Table 2. For Table 1, variables presented show information on dengue data utilized and timeframes covered for historical analysis, types of climate variables assessed, and an overview of findings that include type of health risk estimate, analysis method utilized, and a high-level summary of each study’s finding. Table 2 is similarly structured, with variables indicating type and timing of climate projection scenario utilized, whether the study references changes to mosquito habitat or infection, and a qualitative one-word descriptor summarizing the findings of each study, indicating whether cases are expected to increase, decrease, proceed in a mixed direction, or remain unchanged according to the future projection scenario(s) used in the study. A quality assessment of each article was not undertaken due to the diverse nature of the reviewed material.

**Table 1.** Historical risk of dengue infection based upon climate variables across different regions of the world.

Continent	Publication		Date		Dengue		Climate Indicators					Finding		
	Author	Year	Study area	Time frame	Resolution	Data type	Temperature (°C)	Rainfall (mm)	Relative humidity	Others	Lags	Health outcome	Analysis method	Summarized findings
Asia	Choi et al.	2016	Three provinces in Cambodia	Jan 1998–Dec 2012	Monthly	Cases from National Dengue Control Program	Monthly avg of min, avg, max	Monthly cumulative			0–3 months	IRR (95% CI)	Negative binomial regression	Avg temp and rainfall have significant risk in all three provinces, but inconsistent over 0–3-month lag.
	Lover et al.	2014	Phnom Penh, Cambodia	Sept 2011–Jan 2013	Daily	Lab confirmed	Weekly min	Weekly total	Weekly median		1–15 weeks	IRR (95% CI not provided)	Negative binomial regression	% change in cases is 12–22% increase per 1°C, 0.9–1.3% decrease per mm of rain, and 4% increase per unit RH.
	Fan, Lin et al.	2014	Guangdong Province, China	2005–2011	Daily	Lab confirmed	Daily min, avg, max	Daily total	Daily avg	Daily avg atmospheric pressure, Southern Oscillation Index (SOI)	0–3 days	Excess risk (95% CI)	Time-stratified case-crossover	Daily vapor pressure, avg, and min temps were associated with increased risk; max temp and SOI were negatively associated with transmission; no sig associations for rainfall or humidity.
	Wang et al.	2013	Guangzhou, China	2000–2012	Monthly	Lab confirmed	Monthly avg of min, max	Monthly total	Monthly avg	Monthly avg windspeed	0–2 months	IRR (95% CI)	Zero-inflated Poisson regression	Min temp at 1-month lag and wind speed in the same month had greatest IRR (95%CI) of 2.079 (1.916, 2.256) and 0.048 (0.031, 0.074), respectively. Rainfall at 2-month lag showed negative association. Humidity 1-month lag had 1.10 increase.
	Astuti et al.	2019	Cirebon District, Indonesia	2011–2017	Monthly	Lab confirmed for children 0 to 19 yrs old	Monthly avg	Monthly avg	Monthly avg	Monthly avg NDVI	0–7 months	IRR (95% CI)	Poisson GLM	Avg temp w/ 4-month lag and NDVI w/ 1-month lag had largest IRR (95%CI) with 1.27 (1.22, 1.31) and 3.07 (1.94, 4.86), respectively. Rainfall slight decrease in risk by 1%. humidity at lag 0 month (IRR = 1.05, 95% CI: 1.04–1.06, *P* < 0.001)
	Dhewantara et al.	2019	Bali, Indonesia	2012–2017	Monthly	Clinical diagnosis	Daily avg	Daily total and annual avg	Daily avg			RR (95% CI)	Bayesian spatial Model	RR (95%CI) increased by 1.16 (1.03, 1.31) for each 1-mm increase in rainfall.
	Xu et al.	2019	Bali, Indonesia	2007–2017	Monthly	Clinical diagnosis	Monthly avg of min, avg, max	Monthly total	Monthly Avg	Monthly avg windspeed	0–3 months	RR (95% CI)	Quasi-Poisson w/ distributed lag nonlinear	Avg temp RR (95% CI) increased by 2.95 (1.87, 4.66) per 0.5 °C increase, while risk from rainfall increased by 3.42 (1.07, 10.92) per 7.5 mm.
	Cheong et al.	2013	Three subregions in Malaysia	2008–2010	Daily	Lab confirmed	Daily min, avg, max	Bi-weekly total	Daily avg	Daily avg windspeed (knots)		RR (95% CI)	Poisson GAM	Highest RR (95%CI) were high rainfall of 21.45% (8.96, 51.37), low wind speed of 13.63% (5.42, 34.25), and warm temperature of 11.92% (4.41, 32.19).
	Tuladhar et al.	2019	Chitwan District, Nepal	2010 - 2017	Monthly	Lab confirmed	Monthly avg of min and max	Monthly total	Monthly avg		0–3 months	IRR (95% CI)	Negative binomial regression	Risk increased by more than 1% for increases in min temp (2 month lag), max temp (no lag), and relative humidity (no lag), but decreased by .759% for max temp (3 month lag). No change in risk from rainfall.
	Iguchi et al.	2018	Davao Region, Philippines	2011–2015	Weekly	Clinical diagnosis	Weekly avg	Weekly total		Weekly avg dew point		RR (95% CI)	Quasi-Poisson w/ distributed lag nonlinear	High RR (95% CI) were found for rainfall at 32 mm of 1.697 (1.07, 2.62), dew point at 26°C of 3.10 (1.20, 8.06), temp at 26°C of 1.96 (0.47, 8.15); higher temps (27° to 31°C) had lower RR.
	Benedum et al.	2018	Singapore	2000–2016	Weekly	Lab confirmed	Weekly avg	Excessive rainfall leading to flushing events	Weekly avg		1–20 weeks	OR (95% CI)	Distributed lag nonlinear logistic regression	Significant reduction in outbreak risk 1 to 6 weeks after flushing events. For weeks with 5 or more flushing events, the risk of outbreak in subsequent weeks was reduced by 16 to 70%.
	Struchiner et al.	2015	Singapore	1974–2011	Annual	Reported	Annual avg, min, avg & min combined				1 to 3 (units unclear)	RR (no 95% CI provided)	Poisson GLM	Avg and minimum temperature together explained an RR of 7.1.
	Liyanage et al.	2016	Kalutara District, Sri Lanka	2009–2013	Weekly	Clinical diagnosis	Weekly avg	Weekly total		Running 3-month avg Oceanic Niño Index	0–12 weeks	RR (95% CI)	Poisson time series w/ a two-stage Hierarchical Procedure	Highest RR from rainfall observed at around 10 weeks; linear increase in RR with increasing temperature; RR significantly increasing with ONI more than 0.5 at a lag of 6 months.
	Anno et al.	2015	Northern Region, Sri Lanka	2010–2013	Monthly	Clinical diagnosis	Monthly avg	Monthly avg	Monthly avg			OR (95% CI)	Spatial statistical analysis	Increased OR (95%CI) for rainfall 1.53 (1.418, 1.663) and humidity 1.35 (1.247, 1.461), while protective effect of 0.715 (0.67, 0.762) found for temp.
	Chang et al.	2015	Kaohsiung City, Taiwan	2005–2012	Daily	Lab confirmed	Daily avg	Daily total	Daily avg		2 weeks or 1 month	RR (95% CI)	Poisson regression	Medium/high temp with 2-week lag had negative association, while medium temp w/ 1-month lag had increased RR (95% CI) of 1.32 (1.23, 1.41) and high temp had protective effect of 0.77 (0.71, 0.83); Similar associations for rainfall, while RH had increasing risk with either lag effect.
	Chien et al.	2014	Southern Taiwain	1998–2011	Weekly	Lab confirmed	Weekly min, avg, max	Weekly total, max 24-hr, max 1-hr			1–20 weeks	RR (95% CI)	Distributed lag nonlinear model	RR increased as min temp increased, especially for lag of 5–18 weeks; when max 24-hour rainfall is 50 mm, increased RR lasted for up to 15 weeks; one-month decrease in RR is noted following the extreme rain.
	Phanitchat et al	2019	Northeastern Thailand	2006–2016	Weekly	Clinical diagnosis	Monthly avg of min, max	Monthly avg				IRR (95%CI)	Bayesian Poisson regression	IRR (95%CI) increased by 5.5% (0.9, 11.5%) for every 1 °C of avg max temp increase per month. Mean rainfall and min temp did not have sig risk estimates.
	Wangdi et al.	2018	Timor-Leste	2005–2013	Daily	Clinical diagnosis	Long-term avg annual and seasonal avg	Long-term avg annual and seasonal avg				RR (95% CI)	Multivariate, zero-inflated Poisson regression	RR (95%CI) increased by 0.7% (0.6, 0.8) for 1°C increase in avg temp & 47% (29, 59) for 1 mm increase in precipitation.
	Phung et al.	2018	Vietnam	2005–2015	Monthly	Notified cases	Monthly Avg	Monthly total	Monthly Avg			% change (95% CI)	Multilevel or Zero-inflated negative binomial regression	OR (95%CI) was 5% (3, 7.4) for 1°C increase in avg temp and 15% (13.1, 17) for 1 mm increase in avg rainfall; for every 1% increase in RH a decrease in risk of -3.1% (-3.7, -2.4) was found.
	Lee et al.	2017	Four Provinces in Vietnam	1994–2013	Monthly	Clinical diagnosis	Monthly avg	Monthly total				IRR (95% CI)	GEE w/ auto-regressive	1°C rise in temp increased monthly incidence rate by 13% in Hanoi and 17% in Khanh Hoa; for 100-mm increase in precipitation Khanh Hoa had an 11% increase, An Giang had a 30% and 22% increase in the preceding and same months; Ho Chi Minh City had no significant associations.
	Phung et al.	2016	Mekong Delta Region, Vietnam	2003–2013	Weekly	Clinical diagnosis	Weekly avg	Weekly total	Weekly avg		1–4, 5–8, 9–12 week intervals	RR (95% CI)	Generalized linear-distributed lag models	A 1°C temp increase at lag 1–4 and 5–8 weeks increased RR (95% CI) by 11% (1.09, 1.13) and 7% (1.06, 1.08), respectively; 1% rise in RH increased risk by 0.9% (0.2, 1.4) at lag 1–4 and 0.8% (0.2, 1.4) at lag 5–8 weeks; 1 mm increase in rainfall increased risk by 0.1% (0.05, 0.16) at lag 1–4 and 0.11% (0.07, 0.16) at lag 5–8 weeks.
	Vu et al.	2014	8 provinces in Vietnam	1999–2009	Monthly	Clinical diagnosis	Monthly avg	Monthly total	Monthly avg	Monthly total duration sunshine hours	0–3 months	% change in number of cases (95% CI)	Negative binomial generalized linear models	For Khanh Hoa, Ho Chi Minh, Ca Mau, and Ha Noi % change (95%CI) for every 1% increase in RH was 17.0% (6.8, 28.1), 15.7% (6.0, 26.3), 14.7% (9.5, 20.2), and -24.1% (−35.5, −10.8), respectively; hours of sunshine resulted in −3.9% (−5.4, −2.3), −1.8% (−2.5, −1.1), and 1.6% (0.2, 2.9) for Ha Noi, Ca Mau, and Gia Lai, respectively. For temperature, four provinces had positive increases in risk while 1 province had a protective effect. Rainfall 1 province had increase while another had decrease, others no relationship
	Xuan et al.	2014	Haiphong, Vietnam	2008–2012	Monthly	Surveillance data	Monthly avg	Monthly avg	Monthly avg			RR (95% CI)	Poisson regression	RR (95%CI) was elevated for rainfall (per 50 mm increase) and RH (per 1% increase), with risk being 1.06 (1.00, −1.13) and 1.05 (1.02, −1.08).
	Pham et al.	2011	Dak Lak Province, Vietnam	2004–2008	Monthly	Clinical diagnosis	Monthly avg	Monthly avg	Monthly avg	Monthly avg sunshine hours		RR (95% CI)	Poisson regression	Increased RR (95%CI) for temp (per 2°C increase) of 1.39 (1.25, 1.55), RH (per 5% increase) of 1.59 (1.51, 1.67), and rainfall (per 50 mm increase) of 1.13 (1.21, 1.74); sunshine duration (per 50 hours increase) yielded a protective effect of 0.76 (0.73, 0.79).
Australia	Wenbiao et al.	2012	Queensland, Australia	2002–2005	Daily	Lab confirmed	Monthly avg of max	Monthly avg				RR (95% CI)	Poisson regression	Locally acquired RR (95%CI) increased by 6% (2, 11] and 61% (2, 241) for a 1-mm increase in avg monthly rainfall and a 1°C increase in avg monthly max temp, respectively; overseas-acquired increased by 1% (0, 3) for rainfall.
North America	Brunkard et al.	2008	Matamoros, Tamaulipas, Mexico	1995–2005	Weekly	Lab confirmed	Weekly min, max	Weekly total		Weekly sea surface temperature for Nino 3.4 region	1–18 weeks	% change in dengue incidence (95% CI)	Auto-regressive Model	For 1°C increase in weekly max temp, dengue incidence increased by 2.6% (0.2–5.1) for 1-week lag and by 1.9% (−0.1, 3.9) for a 1 cm increase in weekly precipitation (2-week lag). A 1°C increase in SST resulted in a 19.4% (−4.7, 43.5) increase (18 week lag).
	Moreno-Banda et al	2017	Olmeca Region, Mexico	1995 - 2005	Weekly	Lab confirmed	Weekly min, max	Weekly total		Weekly sea surface temperature for Nino 3.4 region	0–20 weeks	IRR (95% CI)	Negative binomial w/ distributed lags	Statistically significant IRRs were found for 3 of the 10 municipalities per 1°C increase in SST, 6 of the 10 per 1°C increase in min temp, and 5 of the 10 for 1mm increase in rainfall, all with different distributed lags.
	Méndez-Lázaro et al.	2014	San Juan, Puerto Rico	1992 - 2011	Daily	Lab confirmed	Monthly and annual avg of min, max			Monthly avg of sea surface temp, sea level pressure, and windspeed		Factor of transmission increase (95% CI)	Logistic regression	Transmission increased by a factor (95% CI) of 3.4 (1.9, 6.1) for 1°C increase in SST and 2.2 (1.3, 3.5) for min temp over entire period, but increased to 5.2 (1.9, 13.9) for 2007-2011 for SST.
South America	Correia et al.	2017	Arapiraca, Alagoas, Brazil	2008–2015	Monthly	Surveillance data	Monthly avg	Monthly avg	Monthly avg	Monthly avg of dew point temp and windspeed	0–3 months	OR (95% CI)	Logistic regression	Dengue-1 model: highest OR (95%CI) included rainfall-lag1, dew point temp-lag1, and temp-lag1 with a 10.1 (1.4, 73.7), 18.3 (3.6, 93.4), and 26.7 (1.6, 433.1) times greater probability of monthly incidence, respectively. Dengue-2 model: highest OR were temp-lag1 and RH-lag0 of 8.9 and 18.1.
	Limper et al.	2016	Curacao	1999–2008	Monthly	Lab confirmed	Monthly min, avg, max	Monthly total	Monthly avg	Monthly duration sunshine hours	0–8 months	RR (95% CI)	Distributed lag nonlinear model	1°C decrease of avg temp had RR (95%CI) of 17.4% (11.2, 27.0), but a 1°C increase yielded 0.457 (0.278, 0.752); rainfall (per 10-mm increase) yielded 4.1% (2.2, 8.1), maxing out at 6.5% (3.2, 10.0) (1.5 month lag). Low and high humidity have decrease in cases

**Table 2.** Future risk of dengue infection based upon climate projection scenarios across different regions of the world.

Continent	Author	Publication year	Location	Habitat/infections	Projection time frame	Climate scenario utilized	Finding	Projected future direction of dengue
Africa	Mweya et al	2016	Tanzania	Habitat	2020 and 2050	CMIP5	2020 and 2050 climate scenarios show risk intensification in dengue epidemic risk areas with variations across geography.	Increase
Asia	Banu et al.	2014	Bangladesh	Infections	2100	Assessed a 1, 2, and 3.3°C increase in 2100	If temperature increases by 3.3°C, projected increase of 16,030 cases by 2100 in Dhaka.	Increase
	Fan et al.	2019	China	Infections	2020s, 2030s, 2050s, and 2100s	CMIP5 RCP 2.6, 4.5, 6.0, and 8.5	For RCP8.5 in 2100s, the population and expanded high risk areas would increase 4.2-fold and 2.9-fold.	Increase
	Li et al.	2017	City of Guangzhou, China	Infections	2020-2070	CMIP5 RCP 2.6, 4.5, 6.0, and 8.5	Both RCP2.6 and 8.5 have similar trends, but scenario RCP8.5 cases have overall greater incidence.	Mixed
	Ministry of Environment & Forests–Government of India	2012	India	Infections	2030	SRES A1B (temperature and temperature+relative humidity)	In 2030, increase in transmission months in northern areas and reduction in western part of southern India.	Mixed
	Dhiman et al.	2010	India	Infections	2050	HadRM2	With 4°C temperature rise, transmission may be 2 to 5 times more with new areas in northern sub-Himalayan region and in southern most areas.	Increase
	Lee et al.	2018	Korea	Infections	2070	CMIP5 RCP 2.6, 4.5, 6.0, and 8.5	Epidemic duration increases by more than 30 days for RCP 6.0 and 8.5. Vectoral capacity intensity increases more than 2-fold for the RCP 6.0 and 8.5.	Increase
	Sriprom et al.	2010	Sakon Nakhon province in Thailand	Infections	2090-–2099	SRES A1B	Infection spreads from 3 most populated districts to less populated, & transmission period increases from 5 to 9 months.	Increase
Australia	Williams et al.	2016	Queensland cities	Infections	2046–2064	SRES A2 and B1	Decreased dengue transmission predicted under A2, whereas some increases are likely under B1.	Mixed
	Williams et al.	2014	City of Cairns	Habitat	2046–2065	SRES A2 and B1	*A. aegypti* abundance is predicted to increase under B1, but decrease under A2.	Mixed
	Newth et al.	2010	All of Australia	Infections	2030	SRES A1B	Projected cost and DALYs decrease under both mitigation response scenarios that are given across multiple R0 scenarios.	Decrease
	Bambrick et at.	2009	All of Australia	Infections	2020, 2050, 2070, and 2100	Four climate scenarios produced by Australia’s Commonwealth Scientific and Industrial Research Organization	Under ‘no emissions action,’ there is an increase in geographic spread. Under emissions mitigation, transmission-suitable areas remain limited to northern Queensland and to Darwin.	Increase
	Kearney et al.	2009	Northern Territory	Habitat	2010 and 2050	SRES B1	Increased habitat suitability throughout much of Australia; changed water storage practices in response to drought may have greater effect.	Increase
	Teurlai et al.	2015	New Caledonia	Infections	2100	CMIP5 RCP 4.5 and 8.5	Mean incidence rates during epidemics could double if temp rises by 3°C by 2100.	Increase
Europe	Liu-Helmersson et al.	2019	Entire continent and 10-city focus	Habitat	2051–2060 and 2091–2099	CMIP5 RCP2.6 and 8.5	For RCP2.6, minimal change to current situation throughout 21st century, while under RCP8.5 large parts of southern Europe risks being invaded by *A. aegypti*.	Increase
	Liu-Helmersson et al.	2016	All of Europe	Infections	2070-2099	CMIP5 RCP 2.6, 4.5, 6.0, and 8.5	By century end, *A. aegypti* could expand to Northern Europe under RCP8.5. Ae. albopictus could expand to all of Central Europe under RCP8.5; however, would remain the same under RCP2.6.	Increase
	Bouzid et al.	2014	All of Europe	Infections	2011–2040, 2041–2070, and 2071–2100	SRES A1B	Increase in risk projected, with highest incidence rates found for the long-term scenario 2070–2100, with substantial impact for southern Europe.	Increase
	Thomas et al.	2011	All of Europe	Habitat	2011–2040, 2041–2070, and 2071–2100	SRES A1B and B1	Larger parts of the Mediterranean will be at risk. Even some parts of Central Europe (e.g., Southwest Germany) can no longer be excluded at century end.	Increase
North America	Ogden et al.	2014	US and Canada	Habitat	2020s (2011–2040) and 2050s (2041–2070)	CMIP5 RCP 4.5 and 8.5	Modest future northward range expansion of *A. albopictus* by the 2050s, but greater range expansion, particularly in eastern and central Canada.	Increase
	Butterworth et al.	2017	Southeastern USA	Infections	2045–2065	SRES A1B	Mosquito season length in many locations may increase, however projected increases in dengue transmission are limited to the southernmost US locations.	Increase
	Erickson et al.	2012	3 cities in USA	Habitat	2035–2065 and 2069–2099	SRES A1FI and B1	Projected warming shortened mosquito lifespan, which in turn decreased potential dengue season.	Decrease
	Kolivras et al.	2010	State of Hawaii, USA	Habitat	2025–2034	HadCM2	Climate scenarios predict expansion of mosquito habitat and potential dengue risk areas; population at risk projected to go from 532,036 to 1,181,770.	Increase
South America	Cardoso-Leite et al.	2014	Brazil	Habitat	2050	SRES A2a	Area covered by the vector distribution in Brazil will decrease in future projections in the north, but will spread to the south.	Mixed
	Escobar et al.	2016	Ecuador	Habitat	2030, 2050, and 2100	SRES A2	*A. aegypti* potential area of distribution reduced by 69%, 43%, and 48% and population at risk by 84%, 47%, and 40% by 2030, 2050, and 2100, respectively. For *A. albopictus*, the potential area of distribution reduced by 45%, 35%, and 53% and the number of people potentially exposed by 58%, 46%, and 52% in 2030, 2050, and 2100, respectively.	Decrease
	Colon-Gonzalez et al.	2018	Latin America	Infections	2050 and 2100	SSP2 for three different global temperature change scenarios	Number of dengue cases for the 2050s period was 260% larger with about 6.9 million extra cases per year.	Increase
Worldwide	Ryan et al.	2019	Global	Habitat	2050 and 2080	CMIP5 RCP 2.6, 4.5, 6.0, and 8.5	Nearly a billion people could face their first exposure in the worst-case scenario, mainly in Europe and high-elevation tropical and subtropical regions.	Increase
	Messina et al.	2019	Global	Infections	2020, 2050, and 2080	CMIP5 RCP 4.5 SSP1, RCP 6.0 SSP2, and RCP 8.5 SSP3	Do not predict significant spread of dengue risk across continental Europe, with total area at risk increasing from 0.22% in 2015 to 0.62% in 2080, with any expansions in population at risk highly uncertain. Globally, 2.25 billion more people will be at risk of dengue in 2080 compared to 2015, bringing the total population at risk to over 6.1 billion, or 60% of the world’s population.	Mixed
	Campbell et al.	2015	Global	Habitat	2050	SRES A1B, A2, and B1	*A. aegypti* predictions indicate potential for northward expansion in eastern North America, South Asia and East Asia, and southward in Africa and Australia; broadening distributional potential indicated in interior South America and Central Africa. *A. albopictus*, predictions gave clearer indications of expanding distributional potential in eastern North America and East Asia, plus expanding potential across Africa and in eastern and southern South America; distributional potential in Australia was anticipated to expand rather markedly for this species.	Increase
	Rogers	2015	Global	Infections	2080	SRES A1F and B1	A1F models show contraction of distribution in some areas (e.g., Amazon basin) and expansion in others (e.g., southeast African coast & into China).	Mixed
	Proestos et al.	2015	Global	Habitat	2045-2054	SRES A2	Poleward shift of the suitable habitat conditions expected. Significant increase of habitat suitability is projected to occur in eastern Brazil, the eastern US, Western and Central Europe, and Eastern China. Also, significant reductions are projected for northern South America, Southern Europe, Central Africa, Madagascar, and Southeast Asia.	Mixed
	Khormi et al.	2014	Global	Habitat	2030 and 2070	SRES A1B and A2	Contraction in the strongly positive climate areas for *A. aegypti* worldwide.	Decrease
	Hill et al.	2014	Global	Habitat	2030 and 2050	SRES A2	Little-to-no change for A. albopictus in 2030 or 2050.	No Change
	WHO	2014	Global	Infections	2030 and 2050	SRES A1B	Expansion at the fringes of the current distribution of dengue, while socioeconomic developments may counteract this change in most of the world.	Mixed
	Liu-Helmersson et al.	2014	Global	Infections	2070–2099	CMIP5 RCP8.5	Large increases expected by century end in temperate Northern Hemisphere regions.	Increase
	Astrom et al.	2012	Global	Infections	2050	SRES A1B	Economic development can have a large influence on the future risk, with a difference of roughly 0.5 billion people between the highest and the lowest estimate for 2050.	Mixed

## Results

A total of 654 studies (no duplicates) were initially retrieved for screening and assessed for possible inclusion. After exclusion of non-pertinent articles, 30 studies met the final inclusion criteria for providing historical dengue health risk estimates based upon changes in climate variables and 35 studies met the final inclusion criteria for providing future dengue health risk estimates based upon climate projection scenarios (Fig. 1). Most studies were excluded for their irrelevance, particularly around not providing specific health risk estimates or only focusing on model development.

### Historical Health Risk Assessment

**Fig. 2:**
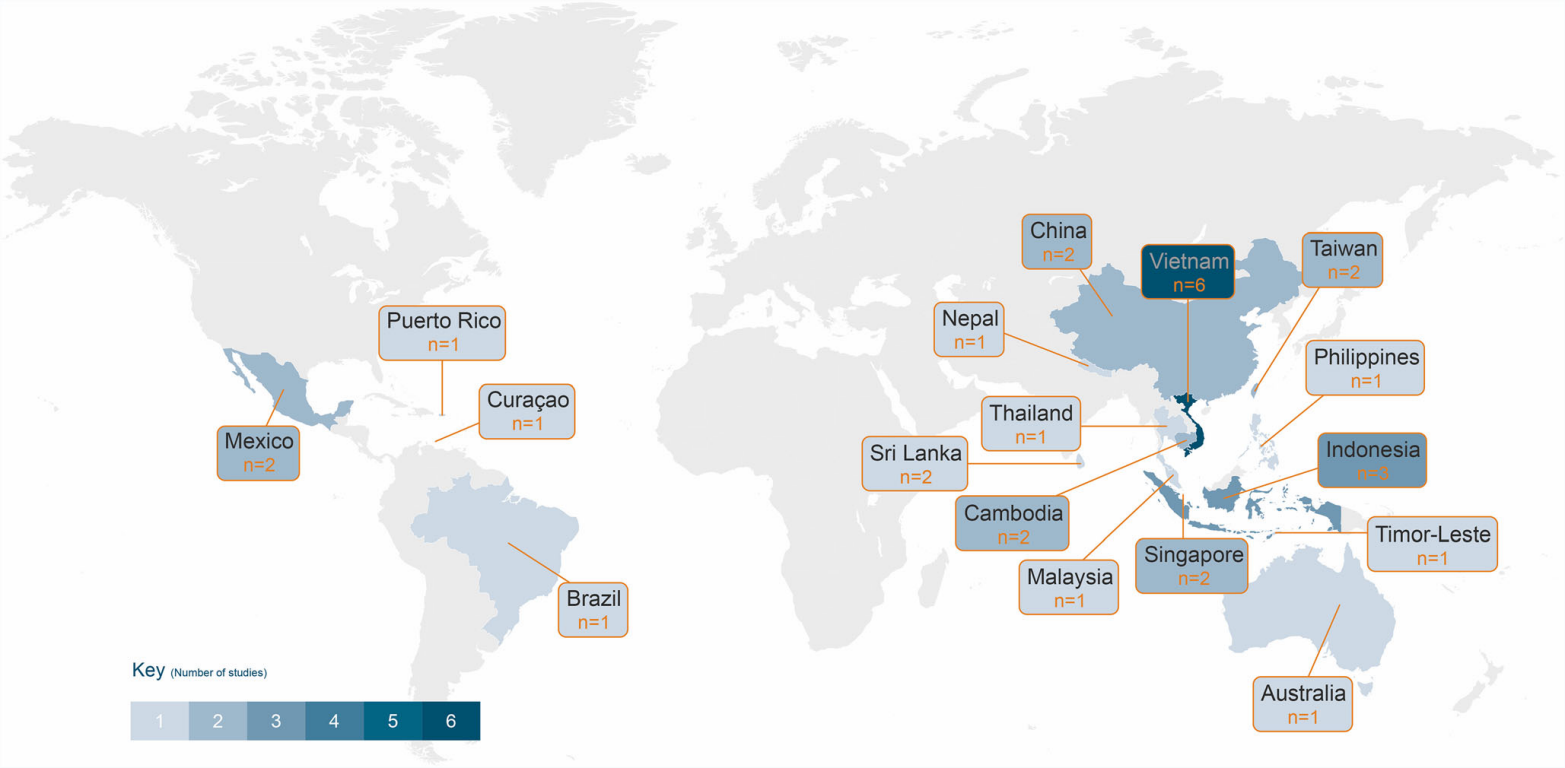
Number of studies assessing historical dengue risk by country

**Fig. 3:**
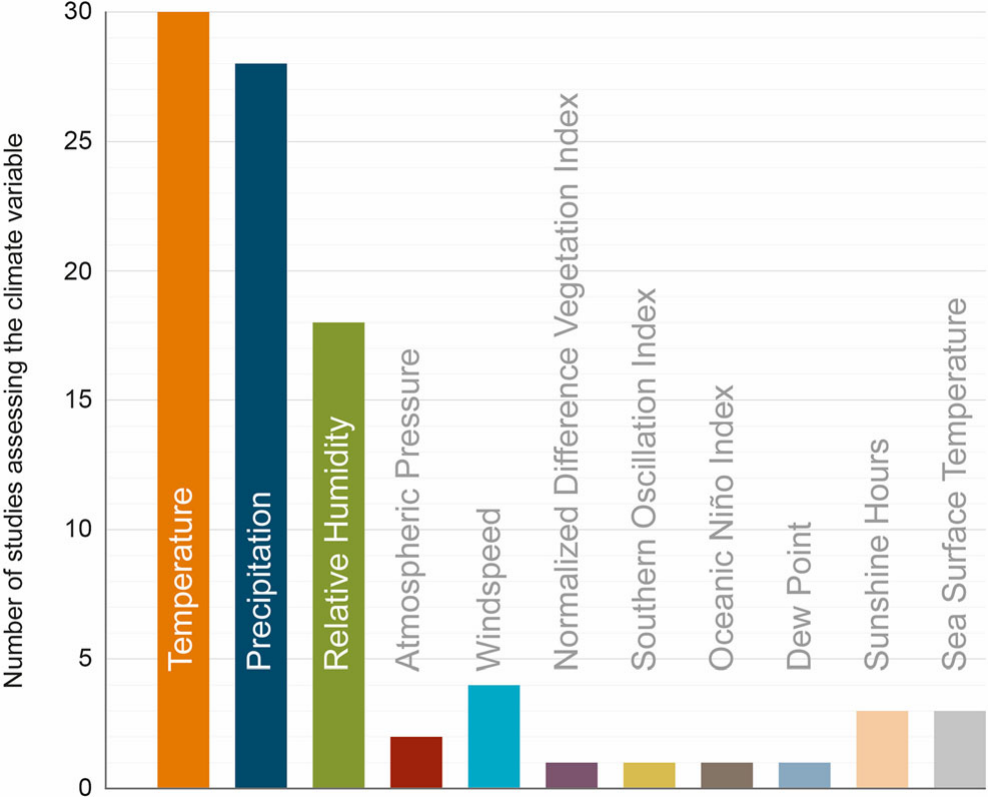
Number of studies assessing the number of dengue cases by climate variable

Nearly all of the 30 studies presenting risk estimates of health impact based upon historical data (see Fig.2) were from the Asian continent (*n*=24), with Vietnam having the most studies from Asia (*n*=6). Air temperature (presented in units of °C), rainfall (in mm), and humidity (%) were the key climate variables (see Fig. 3) used in almost all of the papers, with some papers also including variables such as windspeed (*n*=4), sunshine hours (*n*=3), sea surface temperature (*n*=3), atmospheric pressure (*n*=2), dew point (*n*=2), and normalized difference vegetation index (NDVI, *n*=1). Statistical analysis involved dengue case data that ranged in length from 3 to 28 years on a frequency of daily, weekly, monthly, or annual aggregates that were either clinically diagnosed (*n*=16) or laboratory confirmed (*n*=14). In addition, 18 of the papers included an assessment of lag effects (between climate conditions and dengue cases) that ranged from a few days up to eight months. The studies that were ascertained, based upon the search criteria, developed health risk estimates utilizing a variety of advanced statistical models that included generalized linear models, Poisson and logistic regression, and semiparametric techniques including generalized estimating equations, many of which integrated additional approaches that encompassed negative binomial, nonlinear, or quasi-methods and the incorporation of lag effects. Furthermore, one study by Anno et al. (2015) notably utilized spatial statistical analysis. A summary of significant risk estimates for each study is presented Supplementary information (please see S1: summary table of health risk estimates of dengue infection based upon climate variables).

For developing health risk estimates for contracting dengue based upon changes in temperature (see Table 1), all studies except one [31] included temperature as a part of their assessment. From the 29 studies that conducted health risk assessments as a function of temperature, 19 demonstrated increased risk, seven presented a mixture of increased risk or protective effects, one demonstrated only protective effects, and two studies showed no change in risk. Across the 19 studies showcasing increased risk of dengue infection, differing associations were determined. Highlights include varying increases in incidence of dengue found for every 1°C increase in temperature, from 61% in Australia [32], 12–22% in Cambodia [33], 5% in Vietnam [34], 2.6% in Mexico [35], and 0.7% in Timor-Leste [36]; In Sri Lanka and southern Taiwan, respectively, Liyanage et al. (2016) demonstrated that with increasing weekly average temperature the relative probability of dengue infections increased linearly [37], while Chien et al. (2014) illustrated that dengue risk increased with weekly minimum average temperature especially when accounting for lag effects from the 5 to 18 week range. Of the seven studies showing mixed effects, four studies demonstrated that lower temperatures had increasing risk while higher temperatures had protective effects [38–41]. The risk profile also changed based upon geographic location across almost all of the studies, with Vu et al. (2014) highlighting that even within a country (i.e., Vietnam) risk can increase or decrease based upon location.

For developing health risk estimates for contracting dengue based upon changes in precipitation (see Table 1), 27 of the 30 studies were found to include precipitation as part of their assessment. Of these, 16 studies demonstrated increased risk, five presented a mixture of increased risk as well as protective effects, four demonstrated only protective effects, and three indicated no change in risk. For changes in rainfall, a range of increasing associations of contracting dengue were found including a 47% increase in dengue incidence (per 1 mm increase of rainfall calculated as a seasonal average) in Timor-Leste [36], 5% increase in dengue incidence (per 1mm increase of rainfall calculated as a monthly total) in Vietnam [34], and 6% increase (per 1mm increase of rainfall calculated as a monthly average) in Australia [32]. Multiple studies have shown an increase of chance for contracting dengue with an increase in rainfall in some areas, but no relationships in others depending on geographic location or lag effect utilized [42–46]. Studies have also found a reduction in dengue risk from increasing rainfall. For example, a 0.9–1.3% reduction of dengue cases was found per weekly cumulative mm increase in rainfall in Cambodia [33], a 1% risk reduction per monthly average mm increase in Indonesia [47], and significant reductions in the chance of an outbreak were found due to excessive rain considered to ‘flush’ out mosquito habitats in Singapore [31].

For developing risk estimates of contracting dengue based upon changes in relative humidity, 13 studies were identified. Of these, nine demonstrated increased risk, two studies were found to have a mixture of increased risk as well as protective effects, one study revealed only protective effects, and one study found no association. For a one-unit increase in humidity, a range of increased risk for contracting dengue was found (see Table 1), including increased risk of 4% in Cambodia [33], 10% in China [48], 35% in Sri Lanka [49], and 5% in Vietnam [50]. Additional interesting findings included relative humidity in Curaçao to have a protective effect at either lower or very high levels [51] and changes in the risk profile to be dependent upon geographic location (similar to temperature) even within a country (i.e., Vietnam) [42].

Findings from studies emphasized the importance of temperature, precipitation, and relative humidity, as well as lag effects, when trying to understand how climate change can impact the probability of contracting dengue. Furthermore, studies also emphasized the importance of analyses at a localized level as geographic location can be an important factor in terms of how changes in climate variables can be experienced. This review highlights areas of the world where evidence has been generated and significant areas where risk profiles remain to be developed, particularly for the African continent where no relevant studies were found.

### Future Health Risk Assessment

**Fig. 4:**
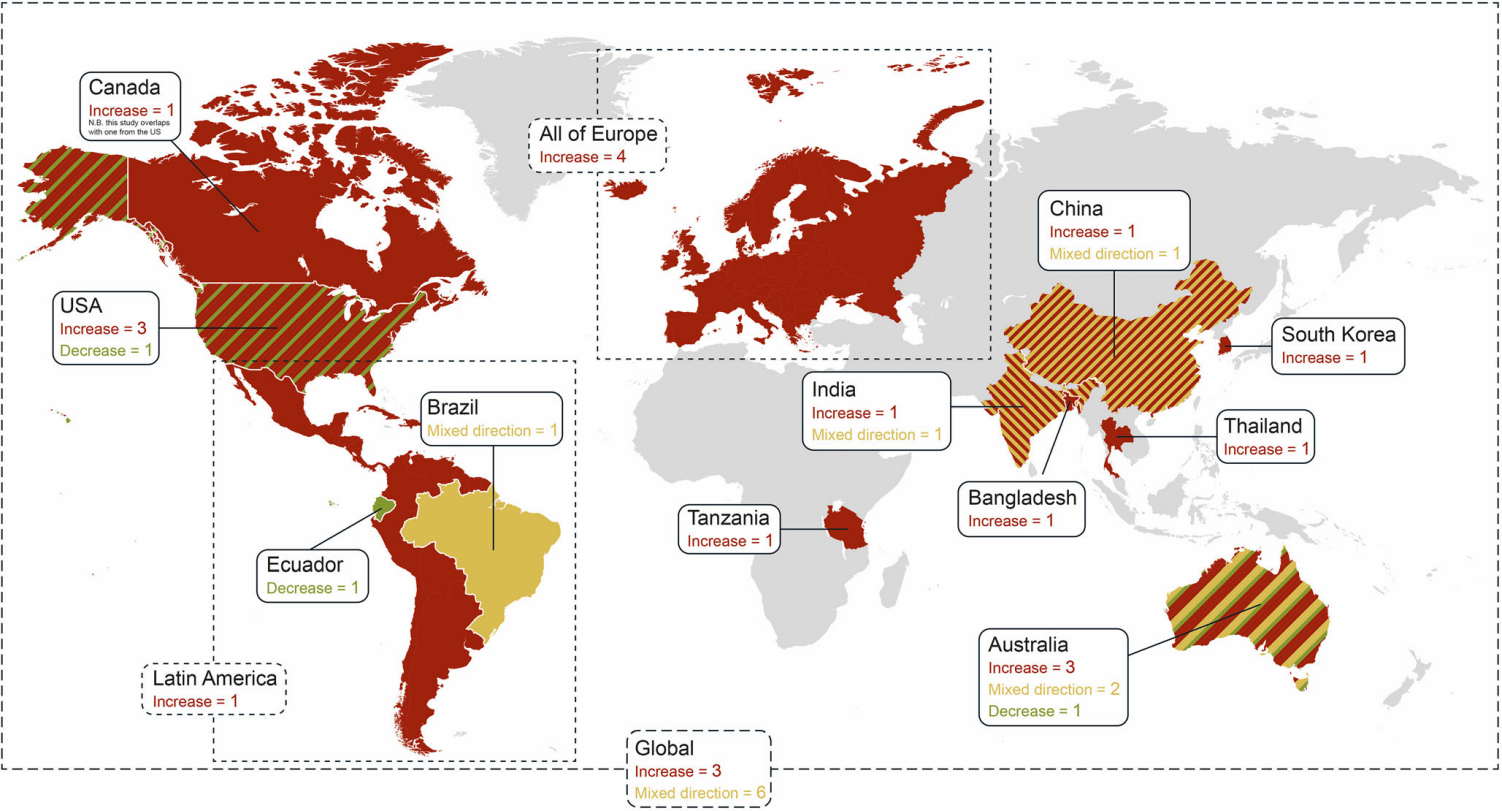
Future changes in the number of dengue cases and the number of studies per country

Of the 35 studies providing health-related risk estimates for contracting dengue based upon future climate projection scenarios extending as far out as the year 2100, 20 studies indicated an increase in future potential for dengue infection, while the others indicated a mixed direction (*n*=11), decrease (*n*=3), or no change (*n*=1) for future dengue infections (see Fig. 4). The majority of the studies (*n*=19) utilized climate projections from the Special Report on Emissions Scenarios (SRES) [52], while others either utilized representative concentration pathway (RCP) scenarios (n=11), which emphasize a greenhouse gas concentration (not emissions) trajectory [53], or a variety of other climate models (*n*=5). Furthermore, 20 of the studies provided health projection estimates based upon historical infection data, while the remaining 15 studies provided infection potential estimates based upon changes in mosquito habitat. In terms of geographic focus, 10 of the studies provided global-level estimates, seven were in Asia, six in Australia, and the rest spread across the remaining continents (excluding Antarctica). Furthermore, the studies developing these future dengue case projection estimates utilized a variety of approaches that encompassed statistical, mechanistic, mathematical, and ecological models.

For the 10 studies that provided only global-level estimates for changes in future dengue case (see Table 2), the number of articles providing changes in the exposure of people to dengue due to changes in mosquito habitat vs. utilizing historical infection data in humans were evenly split (five articles each). Studies utilizing habitat to assess changes in exposure potential provided interesting findings. For example, a study by Ryan et al. (2019) utilized multiple RCP scenarios and projected that for a worst-case scenario by the year 2080 nearly a billion people could face their first exposure to dengue due to changes in mosquito habitat, mainly in Europe and high-elevation tropical and subtropical regions. Campbell et al. (2015) supported the case for an increase in future dengue risk by utilizing SRES A1B (emphasizing rapid economic growth), A2 (emphasizing regionally oriented economic development), and B1 (emphasizing global environmental sustainability) scenarios to predict that by 2050 *A. aegypti* mosquitoes could expand northward in eastern North America as well as in South and East Asia, and southward in Africa and Australia, while also broadening the distribution potential in the interior region of South America and Central Africa [54]. Similarly, Khormi et al. (2014) projected the spread of *A. aegypti* worldwide to contract in the strongly positive climate areas, while currently unfavorable areas, such as inland Australia, the Arabian Peninsula, southern Iran, and some parts of North America may become climatically favorable utilizing the SRES A1B and A2 scenarios. Interestingly, Messina et al. (2019) indicated similar potential expansion as the prior two studies mentioned utilizing the RCP 6.0 scenario, which is known to be similar to the SRES A1B scenario [55]. From a worst-case scenario perspective, Rogers et al. (2015) and Liu-Helmersson et al. (2014) comparatively indicated disagreements for increased numbers of dengue cases by the end of the century utilizing the similar SRES A1F1 and RCP 8.5 scenarios, respectively [55]. Proestos et al. (2015) further highlighted that direction of changes in future potential for dengue infection are geographically dependent, indicating that by the 2050s a poleward shift of the suitable habitat conditions is projected, with a significant increase in habitat suitability to occur in eastern Brazil, USA, China, and western and central Europe, while significant reductions in habitat suitability are projected for northern South America, southern Europe, central Africa, Madagascar, and Southeast Asia. Future global-level projection studies based upon infection data were also highly indicative of the geographic dependence for determining the directionality of dengue infections, with four studies projecting mixed direction and one indicating a clear increase in infections. Noteworthy, Åström et al. [56] and a 2014 report by the World Health Organization [57] indicated that economic development may have a major influence upon the distribution of future dengue risk.

Across the regions of the world, seven studies were conducted in Asia (see Table 2). Bangladesh, Korea, and Thailand each had one study, all indicating increases in dengue potential, with an increase of more than 16,000 cases projected in Dhaka by the year 2100 [58], vectorial capacity increasing by more than 2-fold in Korea by the year 2070 [59], and the transmission period increasing from five to nine months by late century in Thailand [60]. In India, two studies indicated contrasting results, with a report from the Indian government indicating a reduction in transmission by the year 2030 in the southern areas of India [61], while Dhiman et al. (2010) indicated new areas of transmission in southern areas of India by the year 2050. In neighboring China, a study conducted by Fan et al. (2019) indicated that in the 2100s, utilizing the RCP 8.5 (i.e., worst-case) scenario, the population exposed to dengue and expanded high-risk areas would increase by 4.2- and 2.9-fold, respectively. By continent, the second highest number of studies was conducted in Australia (*n*=6). Most of these studies indicated an increase in potential transmission, with Kearney et al. (2009) demonstrating an interesting finding of increased habitat suitability throughout much of Australia by the year 2050, with changes to water storage practices in response to drought as having great influence upon this [58, 60, 62]. Only one study was found to have been conducted on the African continent. Mweya et al. (2016) illustrated that in 2020 and 2050 an intensification in dengue epidemic risk areas is anticipated with variations across Tanzania’s geography.

For the European continent, four studies were found, all of which showed increase in dengue potential, with three of the four studies providing estimates for all of Europe (see Table 2). Studies were largely in agreement, with predictions by century end indicating *A. aegypti* could expand to Northern Europe under RCP 8.5 [63], along with projected increase in dengue cases, with highest incidence rates found for the 2070–2100 timeframe, with substantial impact for southern Europe [64, 65]. The same number of studies (*n*=4) were found to be focused upon the North American continent, with three studies projecting an increase in dengue infection [66–68]. However, a study by Erickson et al. (2012) contrasted these findings, determining that projected warming would shorten the mosquito lifespan thus in turn decreasing the potential for the dengue season.

Lastly, in South America, studies (*n*=3) were found focusing upon Brazil, Ecuador, and Latin America, all indicating a heavy geographic dependence for either the increasing or decreasing probability of contracting dengue (see Table 2). Interestingly, the study focusing on Latin America projected that during the 2050s there would be an additional 6.9 million cases per year, which represents a 260% increase relative to an average yearly number of cases taken from 1961 to 1990 [69].

Nearly a third of the studies from this review showcased global level estimates, with findings across these articles emphasizing the importance of geographical location when trying to assess future risk potential as locations will experience climate change very differently in the years to come. Similar to understanding historical risk at the local level, future level projections are also needed at a localized level so that policy makers can better evaluate how different climate-related measures will influence the chance of dengue outbreaks across their respective regions and, subsequently, concentrate resources in a more targeted and efficient manner.

## Discussion

The findings for both historical dengue outbreaks as well as future projections highlight the many ways that climate change can influence the risk of contracting dengue and therefore its transmission dynamics. Understanding how different climate change variables can influence these dynamics is an important aspect of being able to further investigate these pathways as well as understand potential methods of where interventions can take place. For example, the primary vector of transmission (i.e., the mosquito) has a life cycle that can be impacted by rainfall and temperature [70]. As temperatures rise, this could increase the rate of the development of the mosquito, thereby reducing virus incubation time and resulting in the potential of increased risk of dengue transmission [71–74]. Corollary to this, extreme temperatures have the potential of reducing the mosquito’s ability to survive, thus reducing the potential for transmission of dengue [75]. Precipitation can also influence the development of the vector by creating breeding habitats from standing water after rainstorms that increase transmission risk, or in contrast could result in flushing events from heavy rainfall that could wash away mosquito eggs, thus reducing the transmission potential [76]. Extreme prolonged climatic events can also drive the potential for dengue transmission by influencing human behavior, such as droughts that may result in people increasing water storage practices that could serve as breeding grounds for mosquitos [77]. Broader climate phenomena such as sea surface temperature or Oceanic Niño Index, can influence weather patterns (i.e., changing temperature or precipitation), and depending on the geography can further contribute towards localized impacts as mentioned, thereby altering transmission dynamics [78, 79].

### Future Direction of Research

From the studies reviewed for both historical and future health risk assessment, there are several key areas of research that would benefit from additional focus moving forward. Foremost, it is clear that the impacts of climate change can vary greatly based upon locale, thus impacting the probability of contracting dengue accordingly. Conducting localized health impact assessments (and developing subsequent projections) is needed at the sub-country level so that those in the health sector can develop geographically relevant adaptation measures. Based upon the studies found, more broadly, the European continent is in need of additional research for historical risk assessments, while the African continent is in need for research related to both historical assessments and future dengue case projections. Reviewing the studies revealed limitations, a key one being that many of the historical studies are based on clinical diagnosis (*n*=16 studies), which can be conflated with other diseases that display similar symptoms to dengue. A key area of research requiring attention stemming from this revolves around understanding how climate change impacts the four different serotypes of dengue. Additional research is needed to further understand the risk of historical incidence and future projections of contracting dengue across different serotypes, as well as how this risk changes across different demographic groups and geographic areas. In addition to the health research needed in the realm of how climate change impacts dengue, an improved understanding is also needed around the health–economic impact. Improving upon the understanding of costs associated with the diagnosis and treatment of dengue symptoms can lead to a better understanding of how interventions can benefit from both a health as well as economic lens.

### Addressing Knowledge Gaps

In order to address future areas of research and strengthen the overall understanding of the climate–dengue link, there are several key actions that could serve to address these areas in a more systematic manner. These key actions cover several topics that include building human resource and data architecture capacity, integrating climate–health frameworks into national adaptation plans, and improving engagement with the public. Conducting epidemiological assessments can be a complex process and starts from having reliable data and the capacity to perform such work. Training epidemiologists that are knowledgeable in conducting climate–health risk assessments is an important step in advancing research topics in this realm. Moreover, increasing the functionality of existing data architectures can serve to augment this capacity. Specifically, integrating climate data into existing health systems, along with increasing funding to build lab capacity to conduct more detailed analysis and integrate that data accordingly, is an area that can greatly serve to further advance research on this topic. Given the rapidly changing landscape of dengue-related research, it is also recommended that tools be created to streamline the processes for creating meta-analyses (i.e., integrating health risk estimates across multiple studies for a specific region) given that many countries, particularly low- and middle-income countries, may not have the capacity to continuously assess the scientific literature for updates on risk related research that will have great implications for how they allocate future resources towards the development of adaptation measures. Another action that may serve to advance the climate–health research agenda is to integrate frameworks similar to the US CDC’s Climate-Ready States & Cities Initiative [80] into how ministries of health approach engaging in this issue. By operating from such a framework, greater coordination and a more systematic approach can be utilized to advance research that can be translated into operational interventions. Lastly, seeking to engage the public, such as through the efforts of citizen science, can serve to enhance data sources as well as awareness of the risks and the need for collective action. For example, mobile phones in Tanzania have been utilized to identify mosquito species [81], thereby enhancing entomological data that can increase the ability of researchers to develop a more informed understanding of risk assessment.

Dengue is one of the fastest spreading infectious diseases known, and climate change is a key driver of this change. Possessing an understanding of how climate change impacts the potential for contracting dengue enables the health sector to design robust and localized adaptation measures that span high-level policy response, improved forecasting and early warning systems, resource planning and allocation for health facilities, and communicating with the public. This study provides an overview of the historical and future health risks posed by dengue from climate change and enables the research and policy community to understand where the knowledge gaps are and what areas need to be addressed in order to mitigate the health risks posed by future dengue infection.

## Supplementary Information

ESM 1 (XLSX 16 kb)

## Data Availability

All materials are included in the tables and references provided.
